# Size and molecular weight determination of polysaccharides by means of nano electrospray gas‐phase electrophoretic mobility molecular analysis (nES GEMMA)

**DOI:** 10.1002/elps.201700382

**Published:** 2018-03-25

**Authors:** Victor U. Weiss, Monika Golesne, Gernot Friedbacher, Susanne Alban, Wladyslaw W. Szymanski, Martina Marchetti‐Deschmann, Günter Allmaier

**Affiliations:** ^1^ Institute of Chemical Technologies and Analytics TU Wien (Vienna University of Technology) Vienna Austria; ^2^ Department of Mechanical and Process Engineering University of Kaiserslautern Kaiserslautern Germany; ^3^ Pharmaceutical Institute Kiel University Kiel Germany; ^4^ Faculty of Physics University of Vienna Vienna Austria

**Keywords:** Dextran, Differential mobility analyzer, Gas‐phase electrophoresis, Oat‐ß‐glucan, Pullulan

## Abstract

Size, size distribution and molecular weight (MW) determination of nanoparticles and that are for example large polymers, are of great interest and pose an analytical challenge. In this context, nano electrospray gas‐phase electrophoretic mobility molecular analysis (nES GEMMA) is a valuable tool with growing impact. Separation of single‐charged analytes according to their electrophoretic mobility diameter (EMD) starting from single‐digit EMDs up to several hundred nm diameters is possible. In case of spherical analytes, the EMD corresponds to the dry nanoparticle size. Additionally, the instrument is capable of number‐based, single‐particle detection following the recommendation of the European Commission for nanoparticle characterization (2011/696/EU). In case an EMD/MW correlation for a particular compound class (based on availability of well‐defined standards) exists, a nanoparticle's MW can be determined from its EMD. In the present study, we focused on nES GEMMA of linear and branched, water‐soluble polysaccharides forming nanoparticles and were able to obtain spectra for both analyte classes regarding single‐charged species. Based on EMDs for corresponding analytes, an excellent EMD/MW correlation could be obtained in case of the branched natural polymer (dextran). This enables the determination of dextran MWs from nES GEMMA spectra despite high analyte polydispersity and in a size/MW range, where classical mass spectrometry is limited. EMD/MW correlations based on linear (pullulans, oat‐ß‐glucans) polymers were significantly different, possibly indicating challenges in the exact MW determination of these compounds by, for example, chromatographic and light scattering means. Despite these observations, nES GEMMA of linear, monosaccharide‐based polymers enabled the determination of size and size‐distribution of such dry bionanoparticles.

**Abbreviations**
DMA: differential mobility analyzer
EMD: electrophoretic mobility diameter
GEMMA: gas‐phase electrophoretic mobility molecular analyzer / analysis
GPC: gel permeation chromatography
MW: molecular weight
nES: nano electrospray
OBG: oat‐ß‐glucan
PAMAM: poly(amido‐)amine dendrimer

## 1 Introduction

Today, size, size distribution and molecular weight (MW) determination of nanoparticle material, either (bio‐)organic or inorganic, is a challenging task 1. Also the wide range (e.g. in terms of chemical and physical nature) of macromolecules including genuine and recombinant proteins, polysaccharides, virus particles or DNA share similar challenges. Mass spectrometry (MS) primarily is an excellent method for comprehensive analysis of relatively small compounds. However, it fails in a large number of molecules and particles with a size exceeding the single‐digit nm size range for non‐custom‐built instruments despite native MS is developing toward this size range. In that respect, nano electrospray gas‐phase electrophoretic mobility molecular analysis (nES GEMMA) 2, 3, 4 with a high resolution nano differential mobility analyzer (nano DMA) 5, 6 is a very appealing method. It enables to target analytes from a couple of nanometers up to 200 nm size and above yielding dry nanoparticle size values. Other analytical techniques targeting particles in the same size range are either highly time‐consuming, e.g. microscopic techniques requiring imaging of a larger number of particles to obtain data with good statistics 7 or are biased by larger sample compounds, which are preferentially detected as in light scattering setups employed in batch mode 8, 9.

During nES GEMMA, analytes are electrosprayed from a volatile, aqueous electrolyte solution *via* a cone‐tipped, fused silica capillary. Obtained droplets are dried in a flow of dry particle‐free air and carbon dioxide. Concomitantly, a bipolar ion atmosphere induced by a radioactive source (^210^Po, α‐particle emitter) leads to a steady‐state charge conditioning 10. The dried analytes leaving the nES / ^210^Po chamber after a certain optimized drift time are mostly neutral or single‐charged. They are subsequently sorted according to their electrophoretic mobility diameter (EMD) in a nano DMA applying a well‐defined, tunable electric field and a constant, particle‐free, high laminar air flow. Only single‐charged particles with an EMD correlating to the applied voltage, flow rate, and the DMAs geometry are able to pass the nano DMA unit and are detected by means of a condensation particle counter. There, size‐separated monodisperse nanoparticles are enlarged by means of nucleation in a supersaturated atmosphere (of n‐butanol or water) and subsequently optically detected as they pass a focused laser beam 11. Variation of the applied electric field allows scanning of EMD size ranges in the time scale of seconds to minutes. It is of note that analyte detection is number‐ and not mass‐based. Particle number concentration values are retrieved, as recommended by the European Commission for nanoparticle detection (2011/696/EU from October 11th, 2011).

Kaufman *et al*. showed for the first time a correlation between EMD and MW for a few reference proteins 2. Bacher *et al*. demonstrated the potential of this system for the determination of EMDs for proteins and their oligomers in great detail with deep statistical analysis 12. By comparison of obtained EMDs to MW values, a correlation between these two parameters could be established allowing the MW determination of a protein in question and its specific non‐covalent complexes as well as aggregates from its EMD within this type of compound class. Other works show the applicability of the nES GEMMA system for EMD determination of DNA 13, virus particles (e.g. 12, 14, 15, 16), polyethyleneglycol (PEG) 17, 18 and hyaluronans 19. Wasiak and colleagues 20 employed gas‐phase electrophoresis for the analysis of dextran‐based nanoparticles as had Szymanski and co‐workers 21 much earlier for a small dextran of 11.5 kDa. In the current work, we focus on the application of GEMMA for polysaccharide measurements in a more general approach; we intended to setup an EMD/MW correlation for the wide range of chemically divergent polysaccharides as previously done for proteins and DNA 2, 12, 13, 22, 23.

Poly‐ and oligosaccharides play an important role in a wide field of applications in the pharmaceutical, cosmetic and food industry as active substances, excipients, bulking agents or even blood plasma substitutes 24, 25, 26. We have chosen branched (dextrans) as well as linear glycans (pullulans and oat‐ß‐glucans) for nES GEMMA experiments. Dextrans, mainly produced by *Leuconostoc, Streptococcus* and *Lactobacillus* species, are extracellular, highly‐branched polysaccharides. They consist of α‐(1,6)‐linked glucose units (comprising 50–97% of total linkages) and side‐chains, which are mainly linked *via* α‐(1,3)‐ and to a smaller amount *via* α‐(1,4)‐ or α‐(1,2)‐glycosidic bonds 24. Pullulans are linear biopolymers produced by *Aureobasidium pullulans*. They mainly consist of α‐(1,6)‐linked maltotriose and some maltotetraose units, that is, α‐(1,4)‐glucan chains as typical for amylose, are regularly interrupted by α‐(1,6)‐glycosidic bonds 25. On the other hand, the cellulose‐like linear β‐(1,4)‐glucan chains of oat‐ß‐glucans (OBGs) are interrupted by β‐(1,3)‐linkages and formally consist mainly of cellotriose (58‐72%, three glucose monomers) and cellotetraose units (20‐34%, four glucose monomers) 27. Finally, data for hyaluronans, linear, anionic polysaccharides composed of repeating D‐glucuronic acid and N‐acetyl‐D‐glucosamine units, were found in literature 19 and compared to our results. For a schematic, detailed overview of analyte structures refers to Supporting Information Fig. 1.

## 2 Materials and methods

### 2.1 Chemicals

Six branched dextrans in the mass range of 23.8 to 667.8 kDa and following proteins of different sizes and amounts of glycosylation (refer to Supporting Information Table 1) were purchased from Sigma‐Aldrich (Steinheim, Germany): β‐galactosidase (*E. coli*), enolase (baker's yeast), carbonic anhydrase (from bovine erythrocytes), immunoglobulin G (IgG, bovine), ovalbumin (chicken) and transferrin (human). For calibration with linear polysaccharides, five pullulan standards in the mass range of 22.8 to 788 kDa (provided by S. Alban, Kiel University, Kiel, Germany) and six OBGs in the mass range of 31–1508 kDa (Putus Macromolecular Science & Technology, Wuhan, Hubei, China) were used. Ammonium acetate (≥99.99%) and ammonium hydroxide (ACS reagent) were purchased from Sigma Aldrich (Steinheim, Germany).

### 2.2 Samples and sample preparation

All solutions were prepared by re‐suspending the lyophilized analyte powder in water (18.2 MΩcm resistivity at 25°C) from a Millipore Simplicity apparatus (Billerica, MA, USA). The stock solutions were stored at 4°C. Further dilutions were prepared in 40 mM ammonium acetate (pH 8 ± 0.4) for the analysis of the influence of particle counts on the EMD. For setup of a respective EMD/MW correlation, an aliquot of each analyte from the stock solution was diluted in nine different electrolytes (ammonium acetate −20, 40 and 60 mM each at pH 7 ± 0.4, 8 ± 0.4 and 9 ± 0.4). To assure particle counts did not exceed the limit of 750 particles per detector channel for polysaccharides, dextrans were measured in concentrations of 10 nM (667.8 kDa) to 1600 nM (23.8 kDa), pullulans from 2.5 nM (788 kDa) to 500 nM (22.8 kDa) and OBGs from 50 nM (720 kDa) to 500 nM (31 kDa). The count range for the largest polysaccharide, OBG with 1508 kDa, had to be set to a maximum of 100 particles per detector channel. The concentration range for proteins reached from 25 nM (β‐galactosidase) to 750 nM (carbonic anhydrase). Because the particle counts do not affect the EMD of proteins, it was not necessary to implement a count limit for this class of analytes.

A desalting step was performed for purification of β‐galactosidase samples 28. In addition of measurements of the desalted β‐galactosidase, an aliquot of this sample was heated for 7 min at 50°C to induce dissociation of the biological active form (tetramer) to monomers, dimers and trimers. Sample preparation and measurement occurred on the same day.

### 2.3 nES GEMMA measurements

Our nES GEMMA system (TSI Inc) consists of a nano electrospray (ES) aerosol generator (Model 3480) including a ^210^Po charge reduction device, a nano DMA (Model 3080) and a n‐butanol‐based ultrafine condensation particle counter (Model 3025A). The sheath flow rate inside the nano DMA for all measurements was kept at 15 L/min which allows to separate particles between 2 and 65 nm EMD. To obtain a stable cone jet mode for the nano ES, the voltage was adjusted individually for each sample between 1.7 and 3.0 kV leading to currents of 270–400 nA. The sheath gas flow was set to 1.1 L/min consisting of 1.0 L/min compressed, filtered air and 0.1 L/min CO_2_ (99.5% from Messer, Gumpoldskirchen, Austria). Data were recorded by the *macro*IMS software, v2.0.1 (TSI Inc). Prior measurements, each sample was pre‐sprayed for 3 min (capillary conditioning) by adjusting the pressure in the pressure chamber up to 4.0 psid (pounds per square inch differential, approx. 28 kPa). Rinsing of the fused silica capillary (cone‐tipped, inner diameter of 25 μm, TSI Inc) was performed for at least 3 min previous every conditioning step with the corresponding electrolyte (not including any analyte molecules) in order to remove analyte particles from previous measurements still attached to the inner capillary surface. Obtained data (‘raw counts’) corresponded to the number of analytes recorded for a given EMD (detector channel) and is termed ‘particle counts’. Due to heterogeneous, i.e. polydisperse analytes yielding broad peaks, we refrained to implement further calculated data corrections e.g. for analyte multiple charge probabilities. Every sample was measured seven times (scan time of 120 s) in each of nine different electrolytes, resulting in 63 scans per sample. For each analyte, the median of the seven GEMMA scans (for one electrolyte) yielded a spectrum. The corresponding EMD was determined through fitting of a symmetric Gauss curve to the main analyte peak of the resulting spectrum (OriginPro v 9.1.0, OriginLab, Northampton, MA, USA). The means of all nine EMDs (values from measurements in different buffers) were used for the graphs. For the analysis of the influence of particle counts on the EMD, different concentrations of analytes were measured in 40 mM ammonium acetate, pH 8 ± 0.4 (four scans each, 120 s scan time).

## 3 Results and discussion

The aim of the current work was to demonstrate the feasibility of a nES GEMMA for the analysis of nanometer‐sized polysaccharide nanoparticles of different structure. Based on obtained gas‐phase electrophoresis data, we intended to set up an EMD/MW correlation for highly polydisperse polysaccharides similar to the results reported for proteins 2, 12, PEG with low polydispersity 18 or small (up to 300 kDa, i.e. 11 nm EMD) single‐ and double‐stranded DNA fragments 13. This will allow not only the size determination of dry polysaccharide nanoparticles from nES GEMMA data but also their MW determination.

### 3.1 From proteins to polysaccharides: differences in obtained nES GEMMA spectra

**Figure 1:**
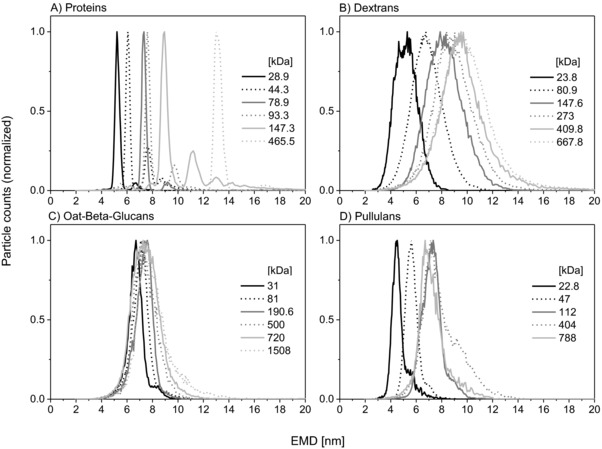
GEMMA spectra of proteins (A), dextrans (B), OBGs (C) and pullulans (D). Each spectrum consists of the normalized median of 63 scans. The spectra of following proteins (A) are plotted: 28.9 kDa ‐ carbonic anhydrase; 44.3 kDa ‐ ovalbumin; 78.9 kDa ‐ transferrin; 147.3 kDa ‐ IgG; 465.5 kDa ‐ β‐galactosidase. For all proteins, the highest peak shows the native form. Protein measurements (A) lead to distinct peaks and multimer species can be distinguished from monomers. Spectra of polysaccharides (B‐C) show broader peaks with increased peak heterogeneity alongside increasing MW values. Dextrans (B) are highly heterogeneous whereas OBGs (C) and pullulans (D) in contrast are less polydisperse.

Spectra for all analytes were recorded in the range of pH 7–9 and 20–60 mM ammonium acetate concentration of sample solutions. Within those values, no influence of these parameters on the observed EMDs of analytes was detected despite an impact of the electrolyte solutions on the size of primary droplets generated during the nES process 29. Therefore, all scans (*n* = 63) were combined to yield EMD values as presented in Supporting Information Table 1. Corresponding nES GEMMA spectra are depicted in Fig. 1.

Protein measurements generally lead to distinct (monodisperse) peaks with the possibility to determine oligomers or multimers of specific and unspecific nature 12. FWHM values of monomer peaks are typically in the range of 0.5 nm for the used instrument. In contrast, nES GEMMA spectra of polysaccharides show broader peaks due to their well‐known natural high polydispersity. For example, Dextran 25 with a company‐provided weight‐averaged MW of 23.8 kDa displayed a FWHM value of 2.1 nm, Dextran 670 with a MW of 667.8 kDa already lead to 4.0 nm FWHM. Polysaccharide peak heterogeneity results from the fact that the sample does not consist of a single analyte species, but (other than for globular proteins which are usually monodisperse, except in case of glycoproteins) of a complex mixture of species with varying size, i.e. a polydisperse sample. These are differing in the number of monosaccharide building blocks (monomers) as larger polysaccharides cannot be separated into monodisperse fractions in larger amounts. Such mixtures are not resolvable by nES GEMMA with standard nano DMAs even at higher flow rates 21. Dextrans displayed the highest FWHM values, whereas OBGs and pullulans turned out to be less polydisperse.

### 3.2 Polysaccharide nES GEMMA spectra interpretation

Broad nES GEMMA peaks together with (i) concentration‐dependent formation of gas‐phase multimers out of statistical reasons during the nES process, (ii) random entanglement of larger analyte chains or (iii) non‐specific analyte interactions in the liquid phase can lead to asymmetric peak broadening due to non‐resolved monomer and multimer peaks. It is of note that these three effects strongly depend on the analyte concentration in the liquid sample. Hence, they can be excluded by measurement of analyte samples at different concentrations. Below a certain concentration threshold, the formation of gas‐phase oligomers and multimers is negligible or absent.

**Figure 2:**
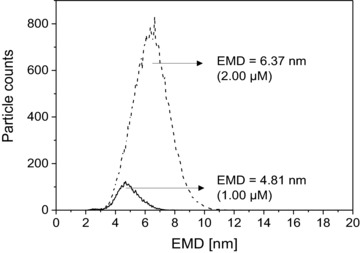
Shift in EMD by increasing particle counts for dextran (23.8 kDa). By doubling the analyte concentration of dextran (23.8 kDa) we could observe a EMD shift from 4.81 nm (approx. 100 particle counts) to 6.37 nm (approx. 750 particle counts). Although the concentration was only doubled, the particle counts were 7.5 times higher than expected. This supports our conclusion that interactions of analytes to the capillary material have to be considered.

If concentration‐dependent multimer formation of polysaccharides during the nES process is ignored, peak apex values are shifted to higher EMDs and may be interpreted as falsely larger diameters of monomer particles. This is demonstrated in Fig. 2 for 23.8 kDa Dextran at 1 μM (slight multimer formation) and 2 μM (increased multimer formation) concentration. However, as long as low‐concentrated samples are analyzed (low interference of non‐resolved multimer peaks with analyte monomers), peak fitting can be applied to obtain an EMD value for the most abundant sample species.

### 3.3 Influence of particle counts on the EMD

The EMD of an analyte can increase with the electrolyte concentration 30 or additional non‐volatile components 31 of a liquid sample. Likewise, the attachment of analyte molecules to the capillary fused silica material has to be considered 32. For the latter, analytes are initially bound to the capillary inner surface and depleted out of solution. After saturation of the surface, the analyte concentration in the liquid phase again increases. However, as polysaccharide EMD values might be biased from unresolved multimer species, the analyte interaction with the ES capillary surface and hence changes in the analyte concentration inside the capillary have to be considered as they might possibly lead to gas‐phase multimerization. As a consequence, we decided to use the unit ‘particle counts’ (i.e. the detector response) as measure for the analyte concentration in the liquid phase inside the nES GEMMA capillary for investigation of this effect: A single 23.8 kDa Dextran sample was measured at different scan times −50, 100 and 200 s for the same EMD range, i.e. the dwell time at each channel of the DMA and hence the overall particle count number was changing. Results did not show an increase in the EMD value of the analyte despite a particle count increase by more than 300% (data not shown). If the particle count value was, however, changing by the same amount using constant scan conditions (e.g. 120 s scan time), we were facing significant shifts in the particle EMD related to unresolved, nES‐induced polysaccharide multimerization.

**Figure 3:**
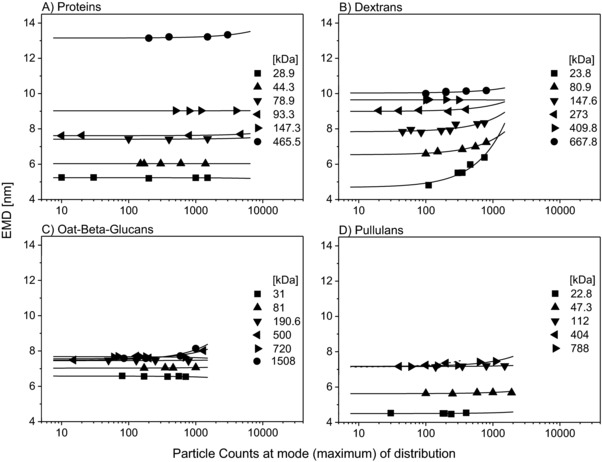
Influence of particle counts on EMD. Each data point constitutes of the median of four scans. Analyte concentrations were chosen that lead to different particle counts. Proteins are not affected in their EMD by the number of detected particles (A). The EMD for dextrans increases with increasing particle counts especially in the lower MW range of 23.8–147.6 kDa (B). An increase in the EMD of OBGs (C) can be observed for analytes of 500 kDa and above. Pullulans also show an increase in EMD at high MWs starting at 404 kDa (D). To demonstrate trends, lines were included in the figure. Especially for analytes measurable only at low particle count rates, the extrapolation has to be regarded with caution (dashed line).

Figure 3 plots particle counts against the EMD for investigated analytes. Proteins are not affected in their EMD by the number of detected particles (see Fig. 3A). Even two‐ to sixfold higher particle numbers (in comparison to polysaccharides) did not change the EMD of proteins significantly due to analyte monodispersity resulting in well‐resolved multimer peaks. In contrast, the EMDs for dextrans were found to increase with increasing particle counts especially in the lower MW range of 23.8 to 147.6 kDa (Fig. 3B). Dextrans with higher MW were less affected. The measured EMD of the selected linear polysaccharides pullulans (Fig. 3C) and OBGs (Fig. 3D) turned out to be considerably less dependent on the particle count than dextrans.

To conclude, particle counts (provided constant scan times are employed) as a measure of the analyte concentration inside the nES capillary play an important role for the determination of the EMD of polysaccharides. Therefore, in order to setup an EMD/MW correlation for polysaccharides, we decided to set a particle count limit of 750 raw counts in order to preferentially detect analyte monomers with our instrument. Spectra with higher particle count numbers were usually not regarded for EMD determination. In case of strongly shifting EMDs (e.g. OBG with a molecular mass of 1508 kDa, i.e. 1.51 MDa) an even lower particle count was chosen to reduce the influence of particle aggregation on EMDs. This approach lead to good statistics as demonstrated by the numbers given in Supporting Information Table 1. Obtained standard deviation values were typically in the range of ±2.6%. It has to be pointed out that the variation of number of scans (contributing to a single spectrum) had no significant effect on observed EMD values indicating that measurements were carried out under steady state conditions.

### 3.4 EMD/MW correlation for polysaccharides

**Figure 4:**
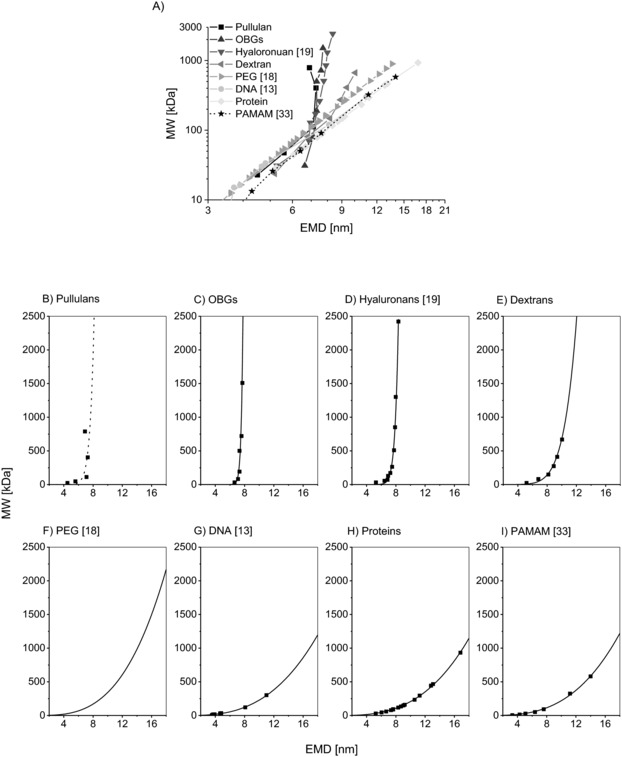
Calculation of EMD/MW correlations. Data points constituting of the median of 63 scans were used to obtain an EMD/MW correlation for different substance classes. Data points are displayed in the logarithmic scale (A) leading to a linear shape EMD/MW dependence for proteins. OBGs, dextrans, pullulans, DNA (data from 13), PEG (correlation function from 18), hyaluronans (data from 19) and PAMAM (data from 33) were plotted as well. Polysaccharide results significantly differed from the EMD/MW correlation for proteins. Above a threshold of approx. 7 nm the EMD does not change significantly for linear polysaccharides (pullulans, OBGs and hyaluronans) with increasing MW. A change in EMD can be observed for branched dextrans even above the 7 nm threshold. The smaller graphs show the non‐logarithmic plots for pullulans (B), OBGs (C), hyaluronans (data from 19, (D), dextrans (E), PEG (correlation from 18, (F) single‐ and double‐stranded DNA (data from 13, (G), proteins (H) and PAMAM dendrimers (data from 33, (I). Curves were fitted to data points, respectively, fit parameters are given in Table 1. Note that due to scattering data points, no fit was possible for pullulans ‐ the displayed dashed curve only reflects a trend.

GEMMA derived EMD/MW correlations for proteins, DNA and PEG have already been described 2, 12, 13, 18, 22, 23. Exemplary data for proteins (own data), DNA (data from Mouradian *et al*. 13) and PEG (correlation from Saucy *et al*. 18) are displayed in Fig. 4 for comparison reasons. By the same token, results for globular shaped poly(amido‐)amine (PAMAM) dendrimers have been published 33 and corresponding data are likewise shown. In case of the PAMAM dendrimers the non‐perfect nature of the synthetic polymeric nanoparticles was shown. With the current study, we aimed to expand the number of available correlations to polysaccharides exhibiting very high polydispersity.

Indeed, we were able to obtain corresponding EMD/MW correlations (Fig. 4). However, polysaccharide behavior differed significantly from proteins, DNA, PAMAM and PEG: Above a threshold of approx. 7 nm EMD (which corresponds to approx. 95 kDa MW on the protein scale), changes in MW for polysaccharides were less reflected in EMD than for proteins. Below the given value, differences between substance classes are not as pronounced (overview in Fig. 4A, individual datasets displayed in Figs. 4B–I). Additionally, it has to be mentioned that we also detected differences for correlations of different polysaccharide classes: linear analytes (pullulans, OBGs) show a different EMD/MW dependency than the branched, more‐bulky dextran. Results from Malm *et al*. for the size determination of linear hyaluronans *via* nES GEMMA 19 are in good agreement (Fig. 4D, based on the mentioned published data) with the results for linear polysaccharides obtained in our work.

In Table 1, fit values can be found for an EMD/MW correlation where the function MW = a × EMD^b^ was fitted to the experimentally determined datasets and literature‐based values for different analyte classes. The value of the exponent of the fit function for DNA, PEG, PAMAM and proteins is approx. 3 which is expected for spheres, or more generally, for sphere‐similar three‐dimensional objects were the axis ratio remains constant. However, for polysaccharides the exponent is considerably exceeding this value.

**Table 1:** Fit values for graphs in Fig. 4B–I; the same fit was applied for all analytes, respectively

	Equation	MW = a × EMD^b^	
	**Adj. R‐Square**	**a**	**b**
B) Pullulans	n. d.	n. d.	n. d.
C) OBGs	0.930	2E‐26 ± 2E‐25	32.654 ± 6.547
D) Hyaluronans (fit for data from ^19^)	0.985	3E‐14 ± 6E‐14	18.408 ± 1.150
E) Dextrans	0.992	7E‐5 ± 7E‐5	6.987 ± 0.459
F) PEG (fit for data from ^18^)	1.000	0.244 ± 0.003	3.146 ± 0.004
G) DNA (fit for data from ^13^)	0.998	0.344 ± 0.064	2.821 ± 0.080
H) Proteins	0.999	0.249 ± 0.021	2.918 ± 0.031
I) PAMAM (fit for data from ^33^)	0.997	0.264 ± 0.074	2.919 ± 0.109

Due to scattering of data points, parameters for pullulans could not be determined (n.d.).

### 3.5 Hypotheses for high exponent values of polysaccharide data fits

**Figure 5:**
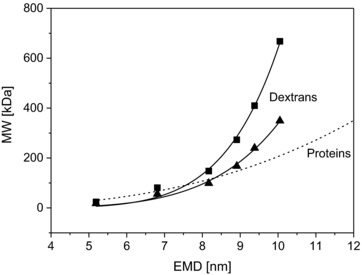
Application of number‐averaged MWs for EMD/MW correlations remedies the deviation of polysaccharide curves from the protein correlation. Especially for dextrans, the modified correlation yields a curve similar to the protein EMD/MW correlation (dashed line). Data based on mass‐averaged MWs (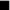) and number‐averaged MWs (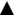) is displayed.

Firstly, (i) solvation problems of sample components with higher MW were excluded as possible cause of the observed polysaccharide EMD/MW correlation deviating from the protein‐based curve. Likewise, (ii) the preferential passage of larger analyte molecules through supposedly at least partially blocked nES capillaries or analyte fragmentation due to low primary droplet size was excluded by variation of the capillary lumen. Further, (iii) the possibility of multiple charge stabilization on polysaccharide particles is highly unlikely as the age of the employed ^210^Po α‐particle source (measurement time points lay several ^210^Po half‐lives apart) did not impact EMD values (data not shown here for all three described cases) and the probability of double of higher number of charges per particle in the investigated size range is known to be rather negligible 10. Another possible explanation of obtained high exponent values concerns the analyte behavior in the gas‐phase: (iv) Polysaccharides with an EMD above the threshold value of approx. 7 nm might not coil up to form spheres during the nES process. Such, the orientation of these analyte nanoparticles in the electric field of the nano DMA is in a way that their EMD on our instrument appears smaller as the Stokes drag force on the particles is lower than for a sphere with the same volume. Particle alignment in a DMA has previously already been described for larger, but very rigid nanoparticles of inorganic nature 34, 35, 36. This hypothesis is additionally supported by the calculated apparent densities of the airborne polysaccharide nanoparticles if a spherical particle shape is assumed (Supporting Information Fig. 2A). Not only that the apparent density of spheres would increase with increasing MW, it would also reach values as usually not found for organic/biological materials (e.g. for pullulan with 788 kDa MW (data provided by the manufacturer) and 7.17 nm EMD (measured) resulting in a density of above 7 g/cm³). Such results are highly unlikely for monosaccharide‐based analytes (note that Kikuchi *et al*. report absolute density values not exceeding 1.51 g/cm³ for amorphous, monodisperse polysaccharides and a decreasing density with a concomitant MW increase for dextrans 37). Therefore, we tried to estimate the nanoparticle axis ratio in approximation under the assumption of an ellipsoid geometry. The measured EMD was set as the diameter of the cross‐section of the cylinder. The density of analytes in the gas‐phase was kept constant at the lowest calculated value (obtained from measurements and suggesting spherical geometry). In doing so, we obtained up to 32‐fold longer particles than suggested by their corresponding EMD (Supporting Information Fig. 2B). Especially for hyaluronans, which are known to exhibit elongated structures 38 such values are high but appear reasonable at least from the biological point of view. In addition, Lin et al. 39 as well as Liu et al. 40 only recently noted the to date not fully understood dynamics of non‐spherical nanoparticles in the gas‐phase. Yet an orientation of the longer axis of an elongated particle parallel to the electric field does not completely explain the observed deviation of the polysaccharides EMD values from the protein case. (v) An additional aspect to be considered is the uncertainty of the provided polysaccharide MW values. The polysaccharide results presented in this manuscript are based on the weight‐averaged MW values as specified by manufacturers and mostly result from gel permeation chromatography (GPC) or light scattering experiments in combination or in stand‐alone fashion. Based on these data also number‐averaged MW values (smaller MW values than upon weight‐averaging) can be calculated and are specified either directly or *via* polydispersity indices (Supporting Information Table 2). In rare cases, e.g. for dextran size standards, additional experiments like end‐group titrations support these calculations. In the absence of such additional experiments and if only weight‐averaged MW values are assessed, smaller sized sample compounds are prone to be overseen as larger particles are preferentially detected. Hence, calculated number‐averaged MW values might still not consider all nanoparticles of a given sample, if no true particle number‐concentration based detection as e.g. permitted by nES GEMMA analysis, is carried out 8. Indeed, if number‐averaged MW values for setting up corresponding MW/EMD correlations are used ‐ i.e. both analyte parameters, EMD and MW, are particle‐number based ‐ the deviation of a polysaccharide from the protein‐based correlation is significantly reduced as demonstrated in Fig. 5 for dextrans. Likewise, the exponent of the correlation function is reduced by about 20% (MW = 7.5 × 10^−4^ × EMD^5.651^; Adj. R‐Square 0.991). In case of linear polysaccharides, calculated number‐averaged MW values still overestimate the MW of analytes by as much as 15–20% as demonstrated *via* MALDI MS in the case of smaller‐sized pullulans (up to 112 kDa) 41. Also, these MALDI MS based data have to be considered with great caution due to the well‐known bias of unfractionated polydisperse samples analyzed by MALDI time‐of‐flight MS. Instrumental constraints did not allow the MS measurement of pullulans of higher MW and particularly of analytes which exhibit higher polydispersity. In addition to challenges in MW determination in the case of linear polymers, also (vi) differences between hydrodynamic (from e.g. GPC) and dry particle diameters (as from nES GEMMA) have to be considered. By the same token, (vii) polymorphism upon crystallization was described for linear synthetic dextrans and might likewise influence the observed EMD upon nES GEMMA analysis on our instrument 42, 43. In sum, all these effects might contribute to the deviation of respective EMD/MW correlations of polysaccharides from the monodisperse protein and DNA particle correlation.

## 4 Concluding remarks

In the present study, we showed for the first time the application of nES GEMMA for the detailed analysis of polydisperse polysaccharide‐based nanoparticles. Protein measurements lead to distinct peaks (FWHM in the range of 0.5 nm) with the possibility to determine multimers due to the fact of their monodispersity. nES GEMMA spectra of polysaccharides on the other hand show broader peaks (FWHM between 2.1 and 4.0 nm which corresponds to relative values of up to 40% of the peak EMD) due to their natural high polydispersity. Nevertheless, peak apices for the main polysaccharide components are well‐defined to be determined from nES GEMMA spectra despite nanoparticle polydispersity allowing their exact size determination. Care has to be taken during experiments to exclude nES induced analyte multimerization as multimer peaks are not fully resolvable (due to the still limited resolution of the applied device) from the main component peak but lead to an EMD shift. No dependency of the measured EMD was found on the ammonium acetate concentration in the range of 20–40 mM or the pH of the ammonium acetate solution in the range of 7.0–9.0.

Furthermore, EMD/MW correlations for polysaccharides were obtained, e.g. in the case of dextrans up to 10.05 nm EMD/332.8 kDa (number‐averaged). Below a size threshold of approx. 7 nm, EMD/MW correlations of all substance classes exhibit a similar behavior. However, above 7 nm EMD, changes in MW for polysaccharides are less reflected in EMD. Additionally, a difference between linear and branched neutral polysaccharide nanoparticles in terms of obtained EMD/MW correlations is observed but cannot be unambiguously explicated. It is of importance for the data evaluation that often only weight‐averaged MWs of reference polysaccharides are provided. The replacement of data by number‐averaged MW values can significantly reduce the deviation of polysaccharide EMD/MW correlations from the other substance classes as shown for dextrans. Additionally, to some extent, directional transport of linear neutral polymers as well as differences between hydrodynamic and dry particle diameters have to be considered besides other effects.

To conclude, in our view the deviation of polysaccharide EMD/MW correlations from protein‐ or DNA‐based correlations originates at least to some extent from imprecise MW determinations of the reference polysaccharide nanoparticles (which are not available in less polydisperse form due to their natural source and the applied isolation/purification procedures). This holds true especially for linear polysaccharides and to a far lesser extend to dextrans. For the latter, an EMD/MW correlation similar in behavior to monodisperse proteins was achieved.

In summary, nES GEMMA is a valuable alternative in the dry size and MW determination of polysaccharide‐based nanoparticles yielding number‐concentration based data and opening up new avenues in monitoring modifications of polysaccharides for nanotechnology, food, cosmetic and pharmaceutical applications 20.

*The authors have declared no conflict of interest*.

## Supporting information

Supporting material
