# Supplementary material for: Size and molecular weight determination of polysaccharides by means of nano electrospray gas‐phase electrophoretic mobility molecular analysis (nES GEMMA)
Source: Electrophoresis. 2018 Mar 25;39(9-10):1142–50. doi: 10.1002/elps.201700382 (PMC6001696; doi:10.1002/elps.201700382)
Supplement: Supplementary file 1 — Supporting material [file ELPS-39-1142-s001.docx]

Electrophoresis (Open Access, to be submitted 2017)

SUPPORTING INFORMATION

| **Victor U. Weiss ^1^**  **Monika Golesne ^1, 2^**  **Gernot Friedbacher ^1^**  **Susanne Alban ^3^**  **Wladyslaw W. Szymanski ^4^**  **Martina Marchetti - Deschmann ^1^**  **Günter Allmaier ^1^**  ^1^ Institute of Chemical Technologies and Analytics, TU Wien (Vienna University of Technology), Vienna, Austria  ^2^ Current address: Department of Mechanical and Process Engineering, University of Kaiserslautern, Kaiserslautern, Germany  ^3^ Pharmaceutical Institute, Kiel University, Kiel, Germany  ^4^ Faculty of Physics, University of Vienna, Vienna, Austria | **Size and molecular weight determination of poly-saccharides by means of nano electrospray gas-phase electrophoretic mobility molecular analysis (nES GEMMA)** |
| --- | --- |

**Corresponding author:** Günter Allmaier, Institute of Chemical Technologies and Analytics, TU Wien (Vienna University of Technology), Getreidemarkt 9/164, A-1060 Vienna, Austria

E-mail: [günter.allmaier@tuwien.ac.at](mailto:martina.marchetti-deschmann@tuwien.ac.at)

Tel: +43 1 58801 15160

Fax: +43 1 58801 15199

**Keywords:** Dextran, Differential mobility analyzer, Gas-phase electrophoresis, Oat-ß-glucan, Pullulan

Supporting information covers an overview on analyte structures (Figure S1) and data concerning density calculations of polysaccharides (Figure S2). Obtained analyte EMD and obtained / company supplied MW values are presented in Table ST1. Table ST2 relates particle polydispersity indices and number-averaged MW values.

**Supplementary Figure S1 – Overview on analyte structures**

**Dextrans**


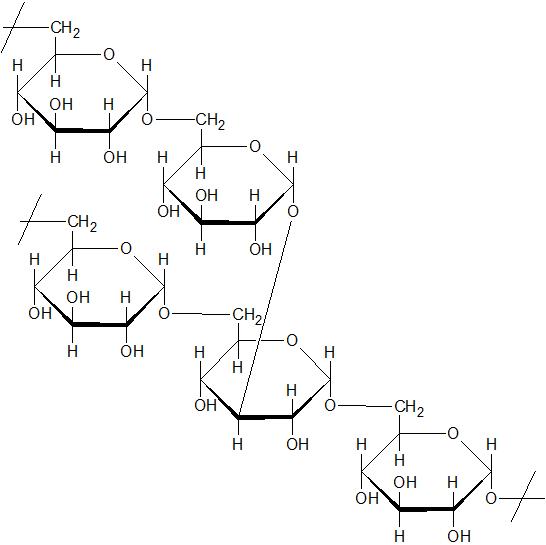


**Pullulans**


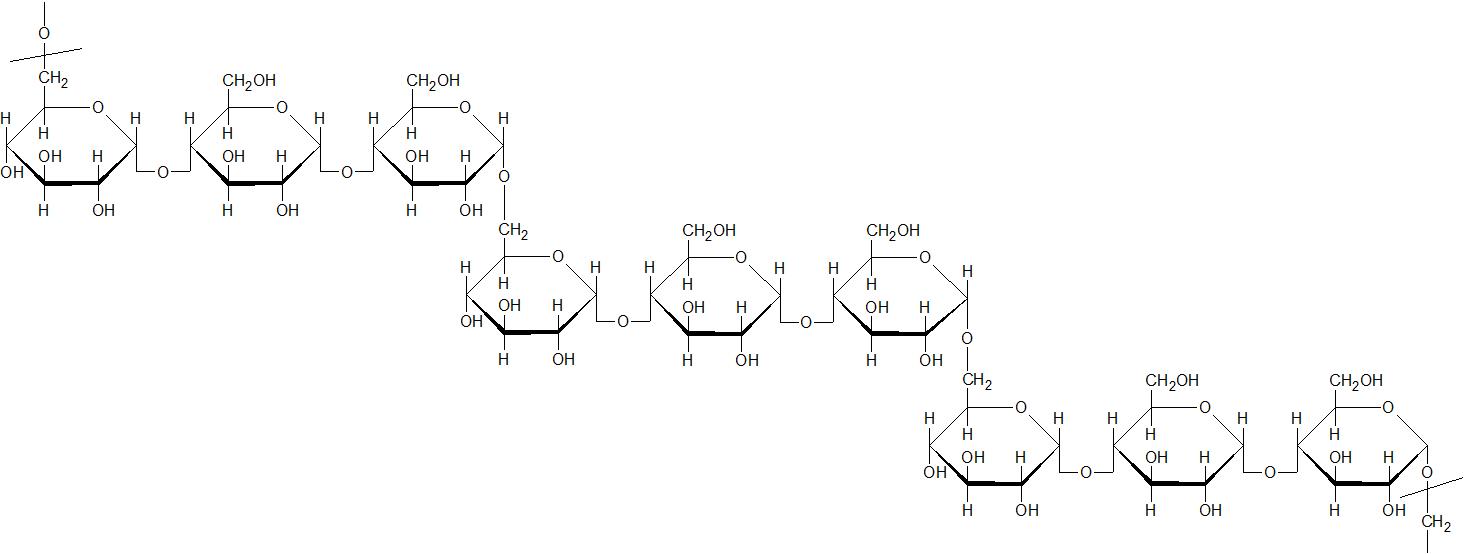


**Oat-β-Glucans**


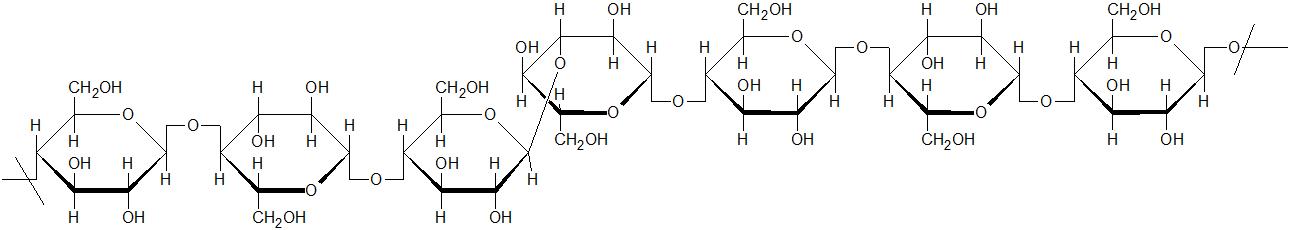


**Hyaluronan**


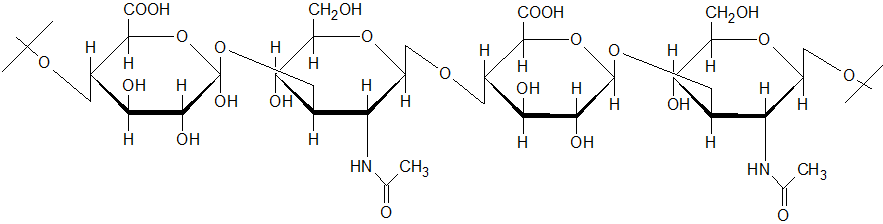


**Supplementary Figure S2 – Calculation of hypothetical analyte densities from nES GEMMA data and resulting deviation of particle shape from spheres.** The graph shows the plot of hypothetical density, calculated assuming a spherical shape of analytes, against their MW (A). Calculations lead to unrealistic high estimated densities for polysaccharides. Figure S2B shows the plot of the axis ratio against the MW. Here we kept the density of analytes in the gas-phase constant (lowest calculated value) and then transformed the shape of the particles in the gas-phase from spheres to ellipsoids. With this correlation we obtained particles up to 32 fold longer than suggested by their corresponding EMD.

**Supplementary Table ST1 – Overview on analyte EMD and MW values;** For proteins the MW of monomers was determined *via* MALDI-MS measurements; for polysaccharides the weight-averaged MW declared by manufacturing companies was taken. The number of measurements consists of the amounts of scans (first position) times the measurements at different conditions (second position). n = measurement number, SD = standard deviation, SD% = percent of standard deviation from mean. For glycoproteins the amount of glycosylation taken from literature (i - iii) is given in brackets.

| **Protein**  **(glycan content**) | **n** | **Form** | **MW [kDa]**  **(from MALDI-MS)** | | **Mean**  **EMD [nm]** | **SD [nm]** | **SD [ %]** |
| --- | --- | --- | --- | --- | --- | --- | --- |
| β-Galactosidase | 7x9 | Monomer | 116.41 ± 0.08 | | 8.33 | 0.11 | 1.28 |
|  | 7x8 | Dimer | 232.82 | | 10.57 | 0.11 | 1.07 |
|  | 7x9 | Tetramer | 465.64 | | 13.08 | 0.05 | 0.38 |
|  | 7x6 | Octamer | 931.28 | | 16.83 | 0.05 | 0.30 |
| Carbonic Anhydrase | 7x9 | Monomer | 28.93 ± 0.01 | | 5.25 | 0.03 | 0.61 |
|  | 7x7 | Dimer | 57.86 | | 6.68 | 0.03 | 0.45 |
| Enolase | 7x9 | Dimer | 93.31 ± 0.03 | | 7.63 | 0.05 | 0.71 |
| IgG (2-3 %) ^i^ | 7x9 | Monomer | 147.27 ± 0.33 | | 9.03 | 0.10 | 1.09 |
|  | 7x9 | Dimer | 294.54 | | 11.26 | 0.10 | 0.92 |
|  | 7x9 | Trimer | 441.81 | | 12.81 | 0.12 | 0.95 |
| Ovalbumin (4%) ^ii^ | 7x9 | Monomer | 44.34 ± 0.01 | | 6.06 | 0.02 | 0.31 |
|  | 7x9 | Dimer | 88.68 | | 7.64 | 0.02 | 0.20 |
|  | 7x9 | Trimer | 133.02 | | 8.71 | 0.02 | 0.19 |
| Transferrin (6%) ^iii^ | 7x9 | Monomer | 78.88 ± 0.36 | | 7.35 | 0.05 | 0.67 |
|  | 7x6 | Dimer | 157.76 | | 9.21 | 0.07 | 0.78 |
|  | | | | | | | |
| **Polysaccharide** | **n** | **Description** | | **MW [kDa]**  **(from company)** | **Mean**  **EMD [nm]** | **SD [nm]** | **SD [ %]** |
| Dextran | 7x9 | 25 | | 23.8 | 5.19 | 0.36 | 6.93 |
|  | 7x9 | 80 | | 80.9 | 6.81 | 0.04 | 0.56 |
|  | 7x9 | 150 | | 147.6 | 8.17 | 0.15 | 1.85 |
|  | 7x9 | 270 | | 273 | 8.91 | 0.24 | 2.71 |
|  | 7x9 | 410 | | 409.8 | 9.38 | 0.42 | 4.50 |
|  | 7x9 | 670 | | 667.8 | 10.05 | 0.41 | 4.12 |
| Oat-β-Glucan | 7x9 | PS-OBG-30 | | 31 | 6.64 | 0.05 | 0.74 |
|  | 7x9 | PS-OBG-80 | | 81 | 7.12 | 0.04 | 0.62 |
|  | 7x9 | PS-OBG-200 | | 190.6 | 7.32 | 0.14 | 1.86 |
|  | 7x9 | PS-OBG-500 | | 500 | 7.32 | 0.25 | 3.35 |
|  | 7x9 | PS-OBG-700 | | 720 | 7.59 | 0.24 | 3.11 |
|  | 7x9 | PS-OBG-H-1500 | | 1508 | 7.71 | 0.15 | 2.02 |
| Pullulan | 7x9 | 22.8 | | 22.8 | 4.50 | 0.09 | 1.95 |
|  | 7x9 | 47.3 | | 47.3 | 5.60 | 0.02 | 0.28 |
|  | 7x9 | 112 | | 112 | 7.12 | 0.13 | 1.83 |
|  | 7x9 | 404 | | 404 | 7.28 | 0.23 | 3.10 |
|  | 7x9 | 788 | | 788 | 6.90 | 0.32 | 4.70 |

i … Arnold, J. N., Wormald, M. R., Sim, R. B., Rudd, P. M., Dwek, R. A., *Annu. Rev. Immunol.* 2007, *25*, 21-50.

ii … Harvey, D. J., Wing, D. R., Küster, B., Wilson, I. B., *J. Am. Soc. Mass Spectrom.* 2000, *11* (6), 564-571.

iii. del Castillo Busto, M. E., Montes-Bayon, M., Blanco-Gonzalez, E., Meija, J., Sanz-Medel, A., *Anal. Chem.* 2005, *77*, 5615-5621.

**Supplementary Table ST2 – Overview on polysaccharide mass- and number-averaged MW values as well as polydispersity indices (PDIs),** Company given values are reported. Matrix assisted laser desorption ionization mass spectrometry (MALDI MS) MW values as found for similar compounds in literature ^iv^ are likewise given. GPC derived values are marked by an asterisk. Calculated values based on company given numbers are highlighted (°).

| **Polysaccharide classes** | **Description** | **MW [kDa]**  **mass-averaged** | **MW [kDa]**  **number-averaged** | **MW [kDa]**  **end-group titration** | **PDI** | **MW [kDa] MALDI MS ^iv^** |
| --- | --- | --- | --- | --- | --- | --- |
| Dextran | 25 | 23.8 * | 18.3 * | 17.94 | 1.30 ° | - |
|  | 80 | 80.9 * | 55.5* | 55.63 | 1.46 ° | - |
|  | 150 | 147.6 * | 100.3* | 98.56 | 1.47 ° | - |
|  | 270 | 273 * | 164.2* | 167.50 | 1.66 ° | - |
|  | 410 | 409.8 * | 236.3* | 239.90 | 1.73 ° | - |
|  | 670 | 667.8 * | 332.8* | 349.30 | 2.01 ° | - |
| Oat-β-Glucan | PS-OBG-30 | 31 | 30.1 ° | - | 1.03 | - |
|  | PS-OBG-80 | 81 | 78.6 ° | - | 1.03 | - |
|  | PS-OBG-200 | 190.6 | 185.0 ° | - | 1.03 | - |
|  | PS-OBG-500 | 500 | 476.2 ° | - | 1.05 | - |
|  | PS-OBG-700 | 720 | 685.7 ° | - | 1.05 | - |
|  | PS-OBG-H-1500 | 1508 | 1396.3 ° | - | 1.08 | - |
| Pullulan | 22.8 | 22.8 | 21.3 ° | - | 1.07 | 17.9 |
|  | 47.3 | 47.3 | 44.6 ° | - | 1.06 | 44.6 |
|  | 112 | 112 | 100.0 ° | - | 1.12 | 87.6 |
|  | 404 | 404 | 357.5 ° | - | 1.13 | - |
|  | 788 | 788 | 640.7 ° | - | 1.23 | - |

iv … Schnöll-Bitai, I., Ullmer, R., Hrebicek, T., Rizzi, A., Lacik, I., *Rapid Commun. Mass Spectrom.* 2008**,** *22*, 2961-2970.
